# Investigating effects of FFP2 wearing during physical activity on gas exchange, metabolism and affective state using a randomized controlled trial

**DOI:** 10.1038/s41598-024-56560-x

**Published:** 2024-03-15

**Authors:** Tobias Engeroff, Katrin Heinsel, Daniel Niederer, Albert Nienhaus, David A. Groneberg, Lutz Vogt

**Affiliations:** 1 Division Health and Performance, Institute of Occupational, Social and Environmental Medicine, Goethe University Frankfurt, Theodor-Stern-Kai 7, Building 9B, 60590 Frankfurt am Main, Germany; 2 Department of Movement and Training Science, Faculty of Humanities and Social Sciences, Institute of Sports Science, University of Wuppertal, Wuppertal, Germany; 3 Institute for Health Service Research in Dermatology and Nursing, University Medical Center Hamburg-Eppendorf, Hamburg, Germany; 4 Institute of Occupational, Social and Environmental Medicine, Goethe University Frankfurt, Frankfurt am Main, Germany; 5 Department of Sports Medicine and Exercise Physiology, Institute of Sport Sciences, Goethe-University Frankfurt, Frankfurt am Main, Germany

**Keywords:** Facemask, Airway resistance, Hypercapnia, Hypoxia, Health care, Medical research, Risk factors

## Abstract

Concerns are repeatedly raised about possible adverse respiratory effects of wearing filtering face pieces (FFP) during physical activity. This study compared the impact of FFP type 2 (NF95) on pulmonary function, blood gas values, metabolism and discomfort during light, moderate and vigorous physical activity. Healthy adults (n = 13; 6 females, 7 males; mean 31.3, SD 5.5 years) participated in this randomized two-armed (Ergometer cycling with a FFP type 2 vs. no mask) crossover trial. Baseline cardiopulmonary exercise testing and two interventions (masked and unmasked ergometer cycling 40%, 50% and 70% VO2max, 10 min each) were separated by 48 h washout periods. Spiroergometric data (End tidal carbon dioxide partial pressure PetCO_2_; breathing frequency; inspiration time), blood gas analysis outcomes (capillary carbon dioxide partial pressure, pCO_2_) and subjective response (Breathing effort and perceived exertion) were contrasted between conditions using ANOVAs. All participants completed the crossover trial, seven started with the FFP2 condition (No adverse events or side effects). FFP2 decreased breathing frequency, prolonged inspiration time, increased perceived breathing effort and PetCO_2_ (*p* < .05). Blood pCO_2_ in millimetres mercury increased during exercise with 50%VO2max (mean 36.67, SD 3.19 vs. mean 38.46, SD 2.57; *p* < .05) and 70%VO2max (35.04, 2.84 vs. 38.17, 3.43; *p* < .05) but not during exercise with 40%VO2max (36.55, 2.73 vs. 38.70). Perceived exertion was not affected (*p* > 0.05) by mask wearing. Conclusion: Mask-induced breathing resistance decreased respiratory performance and limited pulmonary gas exchange. While FFP2 affected subjective breathing effort per se, invasive diagnostics showed that statistically significant metabolic effects are induced from moderate intensity upwards.

*Trial registration*: DRKS-ID: DRKS00030181, Date of registration: 05/09/2022 (German Register for Clinical Trials).

## Introduction

Filtering face pieces (FFP) and masks are commonly used tools to prevent infections both in occupational settings and everyday life. Although such masks limit the risk of airborne infections^1–3^, a continuing discussion about discomfort and potential side effects arose during the Covid-19 pandemic and leads to a reduced public acceptance, in particular during physical activity.

A recently published study included invasive blood gas- and pulmonary gas exchange analysis and indicates that masks might not only alter breathing mechanics but also decrease gas exchange capability during physical work^4^. This adds to the findings of current meta-analyses which relied on a limited number of studies which included invasive measurements. Based on the limited evidence available, meta-analyses suggest a connection between increased breathing resistance and decreased pulmonary function (breathing frequency, tidal volume and ventilation) during exhaustive exercise^5–7^.

Recent research on the impact of FFP and surgical masks during sedentary situations on blood gas concentrations found no impact on invasive measures including oxygen partial pressure (pO_2_), carbon dioxide (pCO_2_), pH, base excess and lactate in healthy subjects^8–11^ and patients with stable chronic heart failure^12^. During this metabolic state (sedentary behaviour), adaptations in breathing patterns such as increased tidal volume or prolonged time for gas exchange due to slower breathing seem thus likely to compensate the influences of increased breathing resistance and additional dead space^5,8–10^. Preliminary evidence however suggests that the impact of FFP and masks during physical activity may follow a linear dose–response relationship and thus could lead to clinically relevant effects at a certain intensity. Four recently published experimental trials suggest an effect on capillary blood pCO_2_ during steady state ergometer cycling with moderate^4^ and vigorous intensity^4,8^ as well as during maximal workload^13,14^ but not during light intensity^4^. One of these studies additionally reported decreased blood oxygen levels that emerged only at high intensity activities^4^. Two other studies on healthy subjects^11^ and patients with chronic heart failure^12^ however were not able to confirm such effects on blood oxygen or blood carbon dioxide concentrations even during maximal workload.

Based on the relative lack of evidence concerning low to moderate intensity activities and the contradictory results concerning adaptations to strenuous physical workload with a mask, further studies are needed to confirm or disprove the impact of FFPs on gas exchange and metabolism. Furthermore, these studies need to analyze more precisely at which intensity mask-induced breathing resistance and a subsequent impairment in respiratory function start to limit carbon dioxide exhalation, blood oxygenation or other clinically relevant metabolic markers. This research is necessary not only to support further application by the general public but also to confirm the risk-free application for medical-, service- and transport personnel during physical labour.

We hypothesize that the suggested increase in breathing resistance during FFP2 wearing has (1) a detrimental effect on breathing performance based on lower breathing frequency (breaths per minute) and prolonged inspiration time (in seconds). Furthermore, we hypothesize that (2) end tidal carbon dioxide partial pressure (PetCO_2_) and (3) carbon dioxide partial pressure in the blood (pCO_2_) are increased during ergometer cycling while wearing an FFP2. Lastly, we hypothesized to find (4) detrimental effects on subjective response to ergometer cycling when compared to no mask wearing.

## Methods

### Study design and ethical aspects

This study has a randomized controlled cross-over design and was approved by the ethics committee of the Department of Psychology and Sports Sciences of the Goethe University (2022-55, approved 30/08/2022). The trial was registered a priori (German Register for Clinical Trials, DRKS-ID: DRKS00030181, date of registration 05/09/2022) and conducted in accordance with the ethical standards set down by the declaration of Helsinki with its recent modification of 2013 (Fortaleza)^15^. To address the knowledge gaps and shortcomings of earlier studies, we conducted a randomized controlled study on the effects of wearing a FFP2 during physical activities of different intensities. To enable blood sampling during- rather than after exercise cessation we applied ergometer cycling. To minimize the confounding influence of air leakage during forced exhalation when a FFP2 is worn under a spiroergometric rubber mask^16^, we analyzed spiroergometric/capnometric measures, such as peak end tidal partial pressures, breathing frequency and the duration of inspiration or exhalation, which are independent of the exhaled air volume as main outcomes for breathing performance and gas exchange.

### Participants

Participants were recruited and the study was rolled out between June and October 2022 in a university in Frankfurt Germany. Eligibility criteria included being from 18 and 50 years of age with no (medical or psychosocial) contraindication against vigorous physical activity. Exclusion criteria were cardiovascular-, pulmonary-, or advanced degenerative musculoskeletal diseases, pregnancy and not completely healed musculoskeletal injury (that affect subjective quality of life or physical performance during exercise).

Sample size calculations were performed based on an earlier study comparing CO_2_ kinetics during steady state exercise with a FFP2 and a surgical mask against a no mask control^8^. A calculation based on an effect size of Cohen’s d = 0.39 (Partial η^2^ 0.136) a significance level of 5% and an 90% power resulted in a sample size of at least 10 participants adopting a crossover design with 6 measurements in repeated measures analysis of variance (rmANOVA). Calculating with a drop-out rate of 20%, a minimum of 12 participants needed to be included in this study.

**Figure 1:**
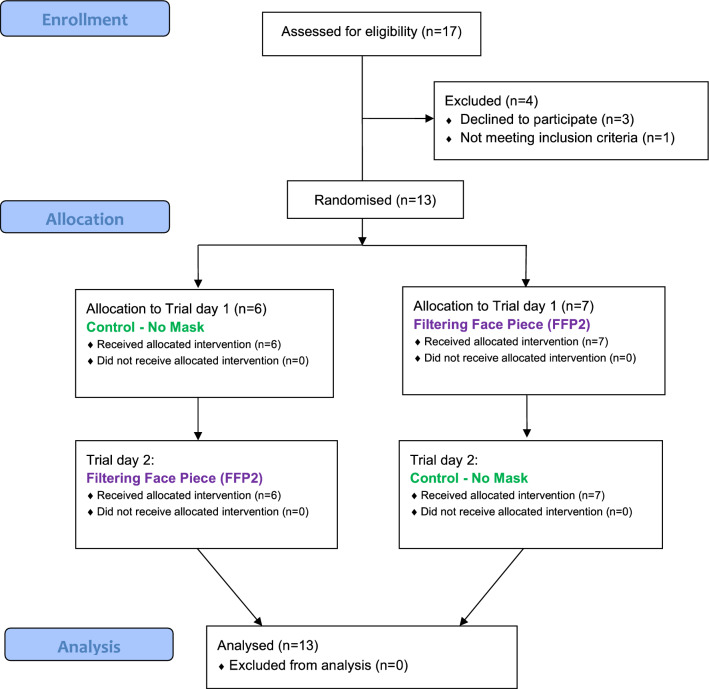
CONSORT flow diagram. Thirteen participants were assigned to either starting with the FFP2 (n = 7) or the unmasked condition (n = 7) by randomisation, followed by crossover to the other condition. CONSORT 2010, Consolidated Standards of Reporting.

Before study participation, participants were informed on voluntary participation and signed a written informed consent. Eligibility, exclusion and randomization scheme of the protocol is shown in the flow diagram in Fig. 1. Order was randomized (simple balanced randomization).

### Interventions

Participants had to avoid vigorous physical activities in the 48 h preceding each test and to maintain their habitual diet during the timeframe of the baseline assessment and both interventions. Furthermore, participants were requested not to take any food or drinks (except for water) during a period of 2 h prior to each examination.

All participants performed a baseline appointment. Two interventions, one with and one without a fold-flat type FFP2 (FFP2 NR, BB203, IMSTec GmbH, Klein-Winternheim, Germany) with three exercise bouts at different intensities each, followed. The duration of each bout was 10 min and the intensities were 40%, 50% and 70% of the individual maximal oxygen uptake (VO2max in milliliters per kilogram bodyweight per minute, ml/kg/min). The baseline assessment and both interventions were separated by a minimum of 48 h. The manipulation order (FFP2 or no mask) was randomized (simple balanced randomization).

Before each intervention, participants rested for five minutes in a seated position without wearing a FFP2. During this time baseline data for spiroergometric outcomes, blood gas analysis and subjective response were assessed. Thereafter the exercise bouts were rolled out in a randomized order. Participants were instructed to pedal in a seated position with at least 60 revolutions per minute and to maintain a comparable pedalling speed for all trials. Between bouts participants rested without wearing a mask for 5 min. Order allocation was done blinded. The participants were blinded to the respective manipulation until the beginning of each intervention. Each trial was performed at a comparable time of the day and at days with comparable routines (i.e. working days).

### Baseline assessment

Assessments at baseline included standard anthropometrical values, educational status (school and study years), habitual physical activity (in Metabolic Equivalent of task hours per week, METh/wk) and sedentary behaviour (International Physical Activity Questionnaire IPAQ)^17,18^ as well as reference values for all assessments described in the outcomes section of this manuscript. Furthermore, cardiopulmonary exercise testing (CPET) until volitional exhaustion was performed at this appointment to assess VO2max and resistance in watts at the point of 40%, 50% and 70% of VO2max. For CPET a ramp shaped protocol (Increment 30 watts per minute for females and 50 watts per minute for males) was rolled out on a cycling ergometer (Optibike, Software OS 1.2, Ergoline GmbH, Deutschland).

### Outcomes

Outcomes included spiroergometric measures, blood gas analysis and subjective data. Spiroergometric measures were taken breath by breath using a wireless system (K5 Wearable Metabolic System, Version 2.0, COSMED GmbH, Werneck, Deutschland) combined with rubber masks with an inspiratory valve covering mouth and nose (V2Mask, Hans Rudolph, Inc. USA). Thirty second floating means were used for analysis. The measuring instrument was calibrated before each test using reference gases (outside air and 5% CO_2_, 16% O_2_) and a standardized ventilatory volume (2 L calibration syringe). The device was tested for sufficient reliability and validity^19^. During cycling interventions rubber masks were either worn with or without a FFP2 underneath. For each subject a tight fit was ensured, and the possibility of escaping air was checked by covering the opening during forced exhalation. Spiroergometric data during interventions were checked for plausibility and analyzed on the basis of a 5 min continuous time block that excluded the first 3 min of each cycling bout to ensure a steady state of metabolism. End tidal carbon dioxide partial pressure (millimeters of mercury), breathing frequency (per minute) and inspiration time (in seconds) were analysed as main outcomes for decreased breathing performance. Additional measures for breathing performance included ventilation (in liters per minute), tidal volume (in liters) and exhalation time (in seconds). Measures for gas exchange were completed by analysing end tidal oxygen partial pressure (in millimeters of mercury) and respiratory exchange ratio (carbon dioxide exhalation divided by oxygen uptake). Heart rate (in beats per minute) was included as outcome for cardiac strain.

For blood gas analysis, capillary blood (100 µl) was drawn from an earlobe of the participant and analysed using a validated on-site device (epoc® Blood Analysis System, Epocal Inc., Ottawa, Ontario, Canada)^20^. Our main outcome for blood gas analyses was carbon dioxide partial pressure (pCO_2_ in millimetres mercury, mmHg). Further outcomes included pH, oxygen partial pressure (pO_2_ in mm/Hg), lactate (in mmol per litre, mmol/l) and base excess (BE in millimoles per litre, mmol/l).

Subjective response included perceived exertion, based on a 15-point Borg Scale ranging from “very very light” (lowest rating 6) to “very very hard” (highest rating 20)^21^, and affective state, based on a 11-point feeling scale ranging from “very bad” (lowest rating − 5) to “very good” (highest rating + 5)^22^. Breathing effort was our main outcomes for subjective response and was analysed using a modified 10-point Borg Category Ratio Scale (Borg-CR) to rate breathing effort ranging from “nothing at all” (lowest rating 0) over “extremely strong” (rating 10) to “maximum” (highest rating “maximum”). As an additional measure for dyspnoea a numeric rating scale for pain ranging from “no pain” (lowest rating 0) to “worst possible pain” (highest rating 10) was applied^23^.

### Data analysis and statistics

We applied Microsoft Excel (Version 16.68) for data processing, SPSS Statistics (Version 29) for imputation of missing data and Prism (Version 9) for data analysis and presentation. Data were analysed as intention-to-treat. Imputation, assuming that missing data was missed completely at random, was made using chained equations and a fully conditional specification model with 40 iterations to produce asymptotically unbiased data estimations. Descriptive data were reported as means with standard deviations (baseline values and post intervention values) or 95% confidence intervals. Differences between baseline values of both trial days and the effect of the manipulation (FFP2 versus no mask) on all outcomes were tested using repeated measures analysis of covariance. Analysis of covariance was applied to evaluate the influence of body mass index, habitual physical activity, smoking status, VO2max and habitual sedentary behaviour on the between manipulations effects.

Lastly, pearson correlations were applied to detect associations between subjective measures, spiroergometric and blood gas analysis data which were affected by FFP2 application. We considered *p* ≤ 0.05 as statistically significant for all statistical analyses.

### Ethical approval

The study design was approved by the ethics committee of the Department of Psychology and Sports Sciences of the Goethe University (2022-55, Approved 2022/08/30).

## Results

### Demographic and baseline data

Thirteen (13) volunteers (6 females, 7 males; mean 31.3, SD 5.5 years) participated in this study and completed the study protocol without any adverse events (Fig. 1). No participant took a break during an exercise bout or stopped cycling prematurely. None of the participants took any medication except oral contraceptives. For seven participants, the first intervention was without a mask and 6 persons started with the FFP2 condition. Demographics, including anthropometric, physical activity and physical performance data, are shown in Table 1. Baseline data of spiroergometric, blood gas and subjective parameters for trials with and without a manipulation (FFP2 and no mask control) are listed in Table 2. No differences between baseline data of both trial days occurred.

**Table 1:** Demographics, self-reported exercise amounts and exercise capacity of our sample.

Outcome	Unit	Mean standard deviation	
**Anthropometric data**			
Age	Years	31.3	5.5
Weight	kg	75.0	13.9
Height	cm	174	6
Body mass index	kg/m^2^	24.6	4.0
**Physical activity via International Physical Activity Questionnaire**			
Vigorous physical activity	MET hours per week	60.0	63.7
Moderate physical activity	MET hours per week	24.2	18.1
Walking	MET hours per week	38.3	53.9
Sedentary behaviour	Minutes per day	272	108
**Maximal values during incremental exercise test**			
Power output	Watt	324	82
Heart rate	Beats per minute	175	11
Oxygen uptake	ml/min/kg	47.2	7.6
Respiratory exchange ratio	without unit	1.28	0.10
Exertion	Borg scale (6–20)	17.8	1.4
Affective response	Feeling scale (− 5–5)	− 0.3	2.1
Breathing effort	Borg CR-10 (0–10)	8.0	2.1
Pain	Numeric rating scale (0–10)	4.4	1.7

**Table 2:** Descriptive data and statistical outputs of baseline values during both intervention days for spiroergometric data (breathing performance, pulmonal gas exchange measures and heart rate), blood gas analysis measures and subjective data. *Indicates significant between-manipulations-effects.

Baseline data (without a mask) on both trial days			
	Mean, standard deviation		ANOVA-output *p* value, eta squared, F value (degrees of freedom)
	No mask	FFP2	
Breathing performamce	Breathing frequency (Breaths per minute)		
	15, 6	15, 5	*p* = 0.954, n^2^ = < 0.001, F(12) = 0.004
	Inspiration time (Seconds)		
	2.1, 1.2	1.9, 0.9	*p* = 0.219, n^2^ = 0.010, F(12) = 1.686
	Exhalation time (Seconds)		
	2.6, 0.9	2.9, 1.8	*p* = 0.468, n^2^ = 0.014, F(12) = 0.561
	Tidal volume (Liters)		
	0.795, 0.272	0.767, 0.361	*p* = 0.703, n^2^ = 0.002, F(12) = 0.152
	Ventilation (Liters per minute)		
	10.7, 2.6	10.1, 2.3	*p* = 0.210, n^2^ = 0.018, F(12) = 1.752
	Heart rate (Beats per minute)		
	70, 10	71, 12	*p* = 0.819, n^2^ = 0.001, F(12) = 0.055
Pulmonal gas exchange	End tidal carbon dioxide partial pressure (Millimeters of mercury)		
	33.4, 3.5	35.0, 3.7	*p* = 0.015*, n^**2**^** = 0.0522, F(12) = 8.001**
	End tidal oxygen partial pressure (Millimeters of mercury)		
	111.7, 4.3	109.5, 4.5	*p* = 0.293, n^2^ = 0.020, F(12) = 1.209
	Respiratory exchange ratio		
	0.88, 0.07	0.89, 0.05	*p* = 0.466, n^2^ = 0.015, F(12) = 0.567
	Carbon dioxide exhalation (Milliliters per minute)		
	301, 63	293, 62	*p* = 0.610, n^2^ = 0.004, F(12) = 0.274
	Oxygen uptake (Milliliters per minute)		
	346, 81.5	329, 70	*p* = 0.357, n^2^ = 0.013, F(12) = 0.919
Blood gas analysis	Carbon dioxide partial pressure (Millimeters of mercury)		
	35.3, 2.7	36.6, 2.3	*p* = 0.052, n^2^ = 0.067, F(12) = 4.677
	Oxygen partial pressure (Millimeters of mercury)		
	85.5, 7.5	88.5, 7.1	*p* = 0.279, n^2^ = 0.046, F(12) = 1.288
	pH value		
	7.44, 0.04	7.43, 0.02	*p* = 0.131, n^2^ = 0.066, F(12) = 2.629
	Base Excess (Millimoles per liter)		
	− 0.44, 1.57	-0.16, 1.54	*p* = 0.386, n^2^ = 0.009, F(12) = 0.810
	Lactate (Millimoles per liter)		
	1.06, 0.30	1.18, 0.53	*p* = 0.635, n^2^ = 0.021, F(12) = 0.635
Subjective data	Perceived exertion (Borg scale 6–20)		
	6.4, 0.7	6.2, 0.4	*p* = 0.190, n^2^ = 0.049, F(12) = 1.929
	Affective response (Feeling scale − 5–5)		
	3.3, 1.4	2.5, 2.1	*p* = 0.147, n^2^ = 0.047, F(12) = 2.410
	Breathing effort (Borg-CR10 scale 0–10)		
	0.4, 0.6	0.4, 0.4	*p* = 0.436, n^2^ = 0.006, F(12) = 0.649
	Perceived pain (Numeric rating scale 1–10)		
	1.2, 0.4	1.2, 0.6	*p* = 0.721, n^2^ = 0.006, F(12) = 0.133

### Spiroergometric data

Descriptive data and the detailed results of the between-manipulations-comparisons for the spiroergometric data are depicted in Table 3. Wearing a FFP2 decreased breathing frequency and prolonged inspiration time during low, moderate and high intensity exercise. Exhalation time was longer solely during low intensity exercise when a mask was applied. Ventilation was lower during low, moderate and high intensity exercise when a mask was worn. Tidal volume was lower only during moderate and high intensity exercise when a mask was applied.

**Table 3:** Descriptive data and Analyses of Variance (ANOVAs with p value, eta squared, F value and degrees of freedom) results for between-manipulations-comparisons (FFP2 vs. no mask) for spiroergometric data (Ventilatory outcomes, pulmonary gas exchange measures and heart rate), blood gas analysis measures and subjective outcomes during low, moderate and high intensity exercise. *Indicates significant between-manipulations-effects.

Between-manipulations-comparison						
	Mean, standard deviation					
	40% VO2max		50% VO2max		70%VO2max	
	No mask	FFP2	No mask	FFP2	No mask	FFP2
Breathing performance	Breathing frequency (Breaths per minute)					
	26.16	22.05	28.09	24.64	32.24	29.75
	4.14	3.73	5.01	3.89	6.21	4.54
	*p* = 0.005*, n^**2**^** = 0.228, F(12) = 11.652**		*p* = 0.005*, n^**2**^** = 0.138, F(12) = 11.957**		*p* = 0.021*, n^**2**^** = 0.054, F(12) = 7.045**	
	Inspiration time (Seconds)					
	1.07	1.35	0.99	1.22	0.91	1.07
	0.24	0.33	0.22	0.26	0.20	0.19
	*p* = 0.018*, n^**2**^** = 0.204, F(12) = 7.524**		*p* < 0.001*, n^**2**^** = 0.195, F(12) = 20.584**		*p* < 0.001*, n^**2**^** = 0.164, F(12) = 35.797**	
	Exhalation time (Seconds)					
	1.37	1.55	1.27	1.34	1.07	1.03
	0.22	0.23	0.21	0.17	0.21	0.16
	*p* = 0.031*, n^**2**^** = 0.145, F(12) = 5.984**		*p* = 0.169, n^2^ = 0.034, F(12) = 2.140		*p* = 0.342, n^2^ = 0.015, F(12) = 0.979	
	Tidal volume (Liters)					
	1.64	1.59	1.91	1.76	2.58	2.22
	0.36	0.69	0.47	0.55	0.76	0.61
	*p* = 0.694, n^2^ = 0.002, F(12) = 0.162		*p* = 0.018*, n^**2**^** = 0.023, F(12) = 7.483**		*p* < 0.001*, n^**2**^** = 0.071, F(12) = 21.199**	
	Ventilation (Liters per minute)					
	41.68	32.56	52.10	41.85	80.27	63.85
	11.25	9.87	15.09	13.29	20.78	13.97
	*p* < 0.001*, n^**2**^** = 0.167, F(12) = 82.333**		*p* < 0.001*, n^**2**^** = 0.123, F(12) = 60.434**		*p* < 0.001*, n^**2**^** = 0.189, F(12) = 32.806**	
Heart	Heart rate (Beats per minute)					
	117.77	120.09	127.99	130.69	156.26	159.15
	13.41	14.49	14.01	12.17	14.55	12.69
	*p* = 0.235, n^2^ = 0.007, F(12) = 1.561		*p* = 0.235, n^2^ = 0.007, F(12) = 1.561		*p* = 0.260, n^2^ = 0.011, F(12) = 1.398	
Pulmonal gas exchange	End tidal carbon dioxide partial pressure (Millimeters of mercury)					
	40.08	42.34	40.39	42.45	40.41	42.97
	4.14	5.04	3.74	3.68	4.03	4.20
	*p*p = .003*, n^**2**^** = 0.061, F(12) = 13.503**		*p* < 0.001*, n^**2**^** = 0.077, F(12) = 24.766**		*p* = 0.009*, n^**2**^** = 0.095, F(12) = 9.806**	
	End tidal oxygen partial pressure (Millimeters of mercury)					
	103.61	101.54	104.56	102.65	107.58	105.38
	4.24	4.87	3.76	3.95	4.69	4.59
	*p* = 0.058, n^2^ = 0.053, F(12) = 4.412		*p* = 0.004*, n^**2**^** = 0.062, F(12) = 12.297**		*p* = 0.008*, n^**2**^** = 0.058, F(12) = 10.044**	
	Respiratory exchange ratio					
	0.88	0.89	0.92	0.93	1.03	1.04
	0.06	0.05	0.04	0.04	0.07	0.08
	*p* = 0.305, n^2^ = 0.015, F(12) = 1.146		*p* = 0.327, n^2^ = 0.023, F(12) = 1.045		*p* = 0.151, n^2^ = 0.013, F(12) = 2.348	
Blood gas analysis Herz-Kreislauf Metabolisch	Carbon dioxide partial pressure (Millimeters of mercury)					
	36.55	38.70	36.67	38.46	35.04	38.17
	2.73	3.44	3.19	2.57	2.84	3.43
	*p* = 0.075, n^2^ = 0.115, F(12) = 3.802		*p* = 0.004*, n^**2**^** = 0.094, F(12) = 12.750**		*p* = 0.024*, n^**2**^** = 0.212, F(12) = 6.673**	
	Oxygen partial pressure (Millimeters of mercury)					
	86.83	86.10	86.12	83.00	90.10	85.57
	7.40	4.52	9.28	6.12	8.53	7.15
	*p* = 0.590, n^2^ = 0.004, F(12) = 0.306		*p* = 0.088, n^2^ = 0.041, F(12) = 3.430		*p* = 0.083, n^2^ = 0.082, F(12) = 3.571	
	pH value					
	7.42	7.42	7.42	7.40	7.35	7.34
	0.02	0.04	0.02	0.03	0.04	0.07
	*p* = 0.851, n^2^ = 0.001, F(12) = 0.037		*p* = 0.035*, n^**2**^** = 0.066, F(12) = 5.640**		*p* = 0.340, n^2^ = 0.024, F(12) = 0.988	
	Base Excess (Millimoles per liter)					
	− 0.75	0.04	− 1.32	− 0.83	− 6.00	− 5.18
	1.56	1.99	2.23	2.81	2.30	3.93
	*p* = 0.055, n^2^ = 0.050, F(12) = 4.527		*p* = 0.344, n^2^ = 0.010, F(12) = 0.972		*p* = 0.515, n^2^ = 0.017, F(12) = 0.451	
	Lactate (Millimoles per liter)					
	1.72	1.40	2.35	2.36	6.41	6.34
	1.37	1.09	1.78	2.04	1.90	2.97
	*p* = 0.171, n^2^ = 0.018, F(12) = 2.116		*p* = 0.969, n^2^ < 0.001, F(12) = 0.002		*p* = 0.923, n^2^ < 0.001, F(12) = 0.010	
Subjective data	Perceived exertion (Borg scale 6—20)					
	9.62	10.62	11.62	11.92	14.77	15.46
	2.63	2.10	2.87	1.94	2.39	2.15
	*p* = 0.053, n^2^ = 0.046, F(12) = 4.588		*p* = 0.600, n^2^ = 0.004, F(12) = 0.291		*p* = 0.201, n^2^ = 0.025, F(12) = 1.834	
	Affective response (Feeling scale -5—5)					
	3.08	1.92	2.69	1.77	1.77	0.00
	1.61	1.55	1.60	1.69	1.79	2.12
	*p* = 0.025*, n^**2**^** = 0.126, F(12) = 6.553**		*p* = 0.033*, n^**2**^** = 0.078, F(12) = 5.799**		*p* = 0.001*, n^**2**^** = 0.181, F(12) = 18.561**	
	Breathing effort (Borg-CR10 scale 0—10)					
	1.73	3.19	2.89	4.15	5.00	7.23
	1.38	1.70	1.66	2.04	1.92	1.96
	*p* = 0.003*, n^**2**^** = 0.194, F(12) = 13.474**		*p* = 0.013*, n^**2**^** = 0.112, F(12) = 8.575**		*p* < 0.001*, n^**2**^** = 0.264, F(12) = 27.424**	
	Perceived pain (Numeric rating scale 1—10)					
	1.54	1.62	1.85	1.62	2.92	2.92
	0.52	1.04	0.69	0.96	1.19	1.26
	*p* = 0.753, n^2^ = 0.002, F(12) = 0.103		*p* = 0.427, n^2^ = 0.020, F(12) = 0.675		*p* = 1.000, n^2^ < 0.001, F(12) < 0.001	

**Figure 2:**
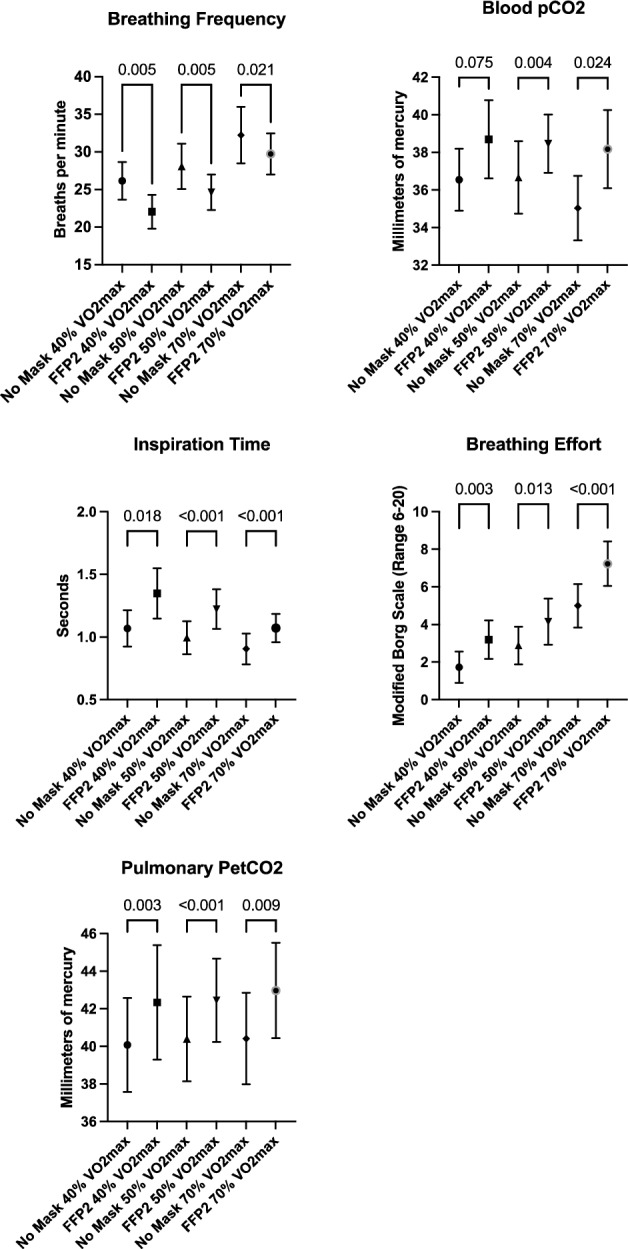
Mean values and 95% confidence intervals of main outcomes including breathing frequency, inspiration time, end tidal carbon dioxide partial pressure (PetCO_2_), partial pressure of carbon dioxide in capillary blood (pCO_2_) and breathing effort during the 40%, 50% and 60% of maximal oxygen uptake (VO2max) cycle ergometer exercise condition with either no mask or a FFP2.

End tidal partial pressure of carbon dioxide was higher during exercise with a mask at all intensities. End tidal pressure of oxygen was lower during moderate and high intensity exercise with a mask. Although respiratory exchange ratio and heart rate values were sensitive to exercise intensity, analyses showed no effects of FFP2 wearing on both outcomes. Figure 2 shows the mean values and 95% confidence intervals of main outcomes including breathing effort, inspiration time and end tidal partial pressure of carbon dioxide. Analysis of covariance showed no impact of smoking status, BMI, habitual physical activity, sedentary behaviour and cardiorespiratory fitness on between manipulations effects for breathing frequency, inspiration time and end tidal pressure of carbon dioxide.

### Blood gas analysis

Descriptive data and the detailed results of the between-manipulations-comparisons for blood gas analysis outcomes of low, moderate and high intensity ergometer cycling are shown in Table 3. Wearing a FFP2 altered carbon dioxide partial pressure and pH during moderate intensity exercise and carbon dioxide partial pressure during high intensity exercise. Figure 2 shows 95% confidence intervals of partial pressure of carbon dioxide which was the main outcome for blood gas analysis. Although there were notable differences between base excess and lactate values during low, moderate and high intensity exercise, blood gas analysis outcomes with and without a mask showed no significant between-manipulations-differences (mask vs. no mask). Analysis of covariance showed no impact of smoking status, BMI, habitual physical activity, sedentary behaviour and cardiorespiratory fitness on between manipulations effects for carbon dioxide partial pressure.

### Self-reported outcomes

Descriptive data and the detailed results of the between-manipulations-comparisons for all subjective outcomes of the walking and stair climbing phase are depicted in Table 3. Participants perceived more breathing effort and reported lower ratings of affective response during ergometer cycling with low, moderate and high intensity when a FFP2 was worn. Figure 2 shows 95% confidence intervals of breathing effort which was the main self-reported outcome. No significant interaction with any tested covariates occurred. Mean values of perceived exertion were higher during more intense exercise but ANOVAs indicated no effect of FFP2 wearing on exertion or pain during all physical activities.

Pearson correlation of breathing effort and affective response with main outcomes of breathing performance, pulmonal gas exchange and blood gas analysis showed no significant associations.

## Discussion

### Hypotheses verification

This study shows preliminary evidence for an effect of FFP2 wearing on breathing frequency and inspiration time during exercise on a cycle ergometer with low, moderate and high intensity. These alterations effect carbon dioxide exhalation indicated by increased end tidal carbon dioxide pressure during all exercise intensities and blood carbon dioxide from moderate intensity onwards. Hypotheses one and two can thus be verified. Hypothesis three can be verified in part. Breathing effort and affective response to exercise were negatively impacted by mask wearing. However, we found no linear association of breathing performance or gas exchange data with both subjective markers. Analyses of perceived exertion and pain showed no between manipulations effects. We thus attest a detrimental effect of FFP2s on subjective breathing effort and affective state but have to reject hypothesis four.

### Mechanisms and effect discussion

The most relevant novelty of our study is the systematic evaluation of the role of exercise intensity using a randomized controlled crossover design and a combination of spiroergometric/capnometric and blood gas analysis measures. Based on this design, we confirmed an effect of FFP2 wearing during low, moderate and high intensity ergometer cycling on breathing performance and gas exchange. Capillary blood gas analysis revealed that these alterations lead to significantly limited carbon dioxide elimination from the blood only from moderate intensity onwards. Further analysis of blood gas data did not indicate significant shifts in the acid base balance nor a significantly decreased blood oxygenation. It thus can be concluded that the cause of the alterations in carbon dioxide concentration is rather a limitation of CO_2_ elimination than an increased production due to limitations in oxygen uptake and subsequent alterations in aerobic and anaerobic energy metabolism.

In contrast to the unaffected blood markers, not only higher carbon dioxide but also a reduced oxygen content was found in the exhaled air. This indicates a higher absorption of oxygen per unit of air and points towards a reduced ventilation. In contrast to the only other study which also compared low, moderate and high intensity exercise and reported a detrimental effect of high intensity cycling on blood oxygenation^4^, cycling with a duration of 10 min with high intensity did not lead to significantly decreased blood oxygen levels in our study. Explanations for these inhomogeneous results can be found in differences in the determination of exercise intensity, in the approach for fitting the FFP2 and the rubber facemask or in the devices for blood gas analysis. Based on a comparison of descriptive data, the mean difference in metabolic outcomes (including pO_2_ and petO_2_) however is comparable between the study of Marek and colleagues^4^ and our data. This confirms, on the one hand, a dose–response relationship between exercise intensity and the effects of mask-wearing, but underlines, on the other hand, an overall low clinical relevance of these effects even at high intensity. Moreover, considering the absence of influence during sedentary behaviour^8–11^ and the contradictory results of blood gas analyses which are performed at the end of a maximum load (some reporting effects on pCO_2_^13,14^ whereas others weren’t able to confirm comparable reactions^11,12^), a rapid compensation of the blood gas alterations after the end of physical activity can be assumed.

In line with current evidence^6^, participants in our experiment perceived increased dyspnoea during exercise with light, moderate and high intensity when a FFP2 was worn. Our results further confirm meta-analytic data which evaluated the effects of different mask types and indicated that although surgical masks have an effect on perceived exertion, a similar effect does not occur with FFP2^6^. It is discussed, that the materials and construction of surgical masks might induce more discomfort due to mask suction and deformation during intense breathing^13^. Against earlier findings on a connection of perceived exertion with breathing frequency and heart rate^24^, we found no linear relation between the subjective and objective effects of FFP2 wearing.

### Limitations and methodological considerations

Two frequently discussed limitations of earlier studies are the application of non-invasive measurements for metabolic data including blood oxygen saturation and the application of spiroergometry to measure the impact of face masks on ventilation. We addressed the first point by applying invasive blood gas analysis instead of pulse derived blood oxygenation. The second point relates to methodological short comings based on a reduction of dead space between the FFP2 mask and the face and on a potential air-leakage between the facial skin, protective mask and rubber-spiroergometric mask which might lead to an underestimation of gross air leakage especially during physical load^16^. To exclude the confounding influence of any possible leakage, we used solely markers that are not dependent on total ventilation volume for our primary outcome measures. However, an explorative analysis of the volumetric measures suggests a negative effect of FFP2s on both, ventilation and tidal volume which tends to increase with exercise intensity. Even assuming undetected measurement errors for these outcomes, the changes in oxygen and carbon dioxide content in exhaled air indicate a decrease in ventilation under the masked exercise condition. A third limitation of our study is the rather small sample size. We calculated the sample size based on the effect of facemask wearing on blood pCO_2_. Therefore, our analysis might underestimate the impact on other outcomes and future studies should further analyze the effects on breathing and oxygen uptake.

### Practical implications for everyday life and research

Our data suggest that FFP2s can be used for up to high-intensity physical activities lasting 10 min or less without risk of clinically relevant hypercapnia or hypoxia. Overall, current research indicates that FFP2s indeed have an impact on breathing and metabolism. However, these effects seem to be rather small and it is likely that the elevation of blood pCO_2_ can be compensated very quickly when the mask is removed during regular breaks. Additional studies however need to evaluate the optimal duration of breaks and the effect of repeated mask wearing. Public information campaigns should highlight that perceived discomfort, breathing effort or dyspnoea is not necessarily linked to negative or even harmful metabolic effects of protective masks.

Despite the rather limited clinical relevance of the acute effects, long-term studies need to analyze the possible impact of elevated CO_2_ levels and increased breathing resistance during long-term mask wearing and on chronic diseases such as hypertension.

## Conclusion

Face masks such as FFP2 induce small changes in pulmonary function and gas exchange during low, moderate and high intensity physical activity. Although blood carbon dioxide levels were affected during moderate and high intensity exercise, invasive metabolic parameters and oxygen values were in a physiological range and did not affect subjective wellbeing. Healthy adults thus seem to be able to fully compensate the impact of mouth and nose protection masks of the FFP2 type during physical activities with low intensity. Consequently, our data underlines that mask wearing in most settings without the option to maintain social distancing over a limited timeframe does not lead to detrimental health consequences. During more intense physical activities FFP2s should be worn over limited timeframes and with regular breaks.

## Data Availability

Data are available upon reasonable request per institutional policy (Contact: Tobias Engeroff, engeroff@sport.uni-frankfurt.de).
